# μ-Oxalato-bis­[bis­(2,2′-bipyridine)­manganese(II)] bis(perchlorate) 2,2′-bipyridine solvate

**DOI:** 10.1107/S1600536811038475

**Published:** 2011-09-30

**Authors:** Kang-kang Li, Chun Zhang, Wei Xu

**Affiliations:** a Center of Applied Solid State Chemistry Research, Ningbo University, Ningbo, Zhejiang, 315211, People’s Republic of China

## Abstract

The unit cell of the title compound, [Mn_2_(C_2_O_4_)(C_10_H_8_N_2_)_4_](ClO_4_)_2_·C_10_H_8_N_2_, consists of a binuclear cation, two perchlor­ate anions, and one solvent 2,2′-bipyridine (bpy) mol­ecule. In the complex cation [Mn_2_(C_2_O_4_)(C_10_N_2_H_8_)_4_]^2+^, two Mn^II^ atoms are bridged by a bis­(bidentate) oxalate ligand, each Mn^II^ atom being further coordinated by two bpy ligands in a distorted octa­hedral geometry. The distance between the two six-coordinated metal atoms is 5.583 (1) Å. π–π stacking inter­actions [inter­planar distances between bpy rings = 3.739 (1) Å] are essential to the supramolecular assembly. There are extensive inter­ionic C—H⋯O inter­actions between the cations and between the cation and anion. Three of the four perchlorate O atoms are disordered over two sets of sites with occupancy ratios of 0.852 (6):0.148 (6).

## Related literature

For general background to π–π inter­actions, see: Janiak (2000 ▶). For structures containing similar cations, see: Chen *et al.* (2005 ▶); Jurić *et al.* (2007 ▶); Sun *et al.* (2009 ▶). 
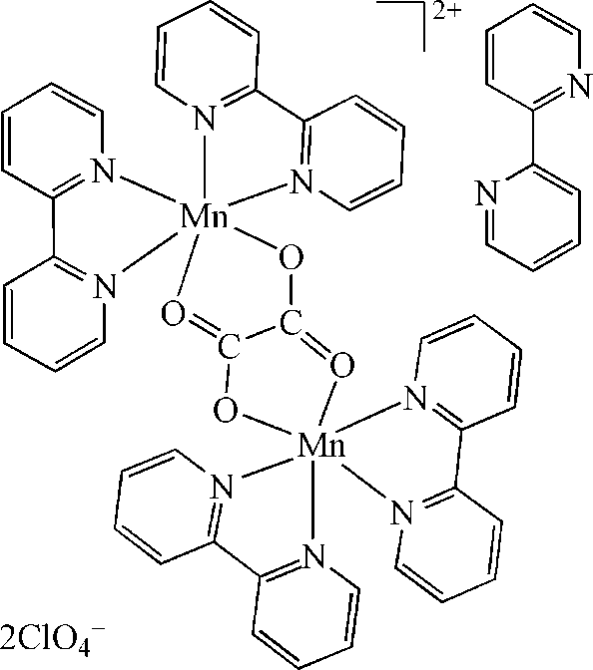


## Experimental

### 

#### Crystal data

[Mn_2_(C_2_O_4_)(C_10_H_8_N_2_)_4_](ClO_4_)_2_·C_10_N_2_H_8_
*M* *_r_* = 1177.72
Triclinic, 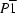

*a* = 9.4959 (19) Å
*b* = 11.974 (2) Å
*c* = 12.183 (2) Å
α = 98.87 (3)°
β = 102.73 (3)°
γ = 98.89 (3)°
*V* = 1309.3 (4) Å^3^
*Z* = 1
Mo *K*α radiation
μ = 0.66 mm^−1^
*T* = 293 K
0.18 × 0.15 × 0.08 mm

#### Data collection

Rigaku R-AXIS RAPID diffractometer
Absorption correction: multi-scan (*ABSCOR*; Higashi, 1995 ▶) *T*_min_ = 0.888, *T*_max_ = 0.949
12924 measured reflections
5884 independent reflections
2595 reflections with *I* > 2σ(*I*)
*R*_int_ = 0.071

#### Refinement

*R*[*F* ^2^ > 2σ(*F* ^2^)] = 0.072
*wR*(*F* ^2^) = 0.221
*S* = 1.06
5884 reflections
363 parameters
19 restraints
H-atom parameters constrained
Δρ_max_ = 0.56 e Å^−3^
Δρ_min_ = −0.64 e Å^−3^

### 

Data collection: *RAPID-AUTO* (Rigaku, 1998 ▶); cell refinement: *RAPID-AUTO*; data reduction: *CrystalStructure* (Rigaku/MSC, 2004 ▶); program(s) used to solve structure: *SHELXS97* (Sheldrick, 2008 ▶); program(s) used to refine structure: *SHELXL97* (Sheldrick, 2008 ▶); molecular graphics: *SHELXTL* (Sheldrick, 2008 ▶); software used to prepare material for publication: *SHELXL97*.

## Supplementary Material

Crystal structure: contains datablock(s) ptcLa, I. DOI: 10.1107/S1600536811038475/bv2191sup1.cif

Structure factors: contains datablock(s) I. DOI: 10.1107/S1600536811038475/bv2191Isup2.hkl

Additional supplementary materials:  crystallographic information; 3D view; checkCIF report

## Figures and Tables

**Table 1:** Hydrogen-bond geometry (Å, °)

*D*—H⋯*A*	*D*—H	H⋯*A*	*D*⋯*A*	*D*—H⋯*A*
C5—H5*A*⋯O1^i^	0.93	2.61	3.356 (6)	138
C5—H5*A*⋯O2^ii^	0.93	2.44	3.243 (6)	144
C10—H10*A*⋯O3^iii^	0.93	2.63	3.484 (6)	153
C18—H18*A*⋯O5*A*	0.93	2.46	3.360 (8)	162
C23—H23*A*⋯O4*A*	0.93	2.64	3.309 (7)	129
C11—H11*A*⋯O6*B*^iv^	0.93	2.52	3.373 (10)	152
C14—H14*A*⋯O5*B*^v^	0.93	2.72	3.243 (12)	117
C18—H18*A*⋯O5*A*	0.93	2.46	3.360 (8)	162
C18—H18*A*⋯O5*B*	0.93	3.18	4.069 (10)	161
C19—H19*A*⋯O6*B*	0.93	2.69	3.225 (9)	117

Symmetry codes: (i) 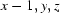; (ii) 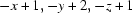; (iii) 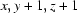; (iv) 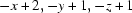; (v) 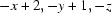.

**Fig. 1.**
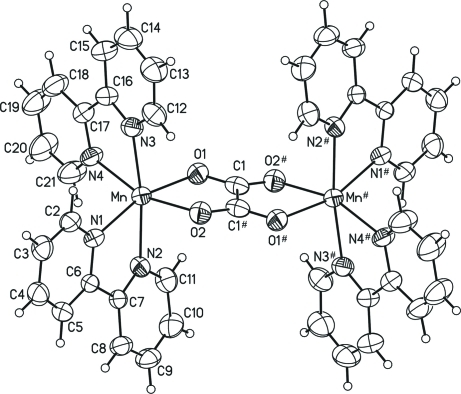
ORTEP view of the binuclear cation. The displacement ellipsoids are drawn at the 35% probability level.

**Fig. 2.**
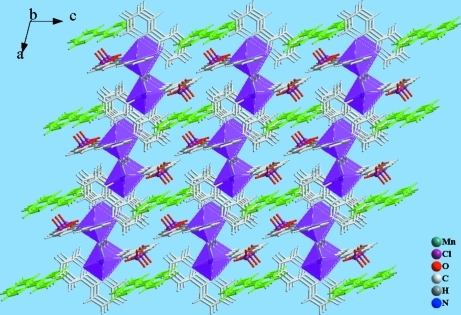
Packing diagram of the title crystal structure viewed along [010] direction.

## supplementary crystallographic information

### Comment

Herein, we report a new oxalate-bridged Mn(II) complex namely [Mn_2_(C_10_N_2_H_8_)_4_(C_2_O_4_)](ClO_4_)_2_.(C_10_N_2_H_8_). The unit cell of the title compound consists of a binuclear cation, two perchlorate anions, and one solvent 2,2'-bipyridine (bpy) molecular. The cationic unit is similar to those seen in analogous Zn (Sun *et al.*, 2009), Cu (Jurić *et al.*, 2007), and Cr (Chen *et al.*, 2005) complexes. As shown in Fig. 1, the complex cation [Mn_2_(C_10_N_2_H_8_)_4_(C_2_O_4_)]^2+^ has two manganese metal centres bridged by a planar bis(bidentate) oxalate ligand, with the coordination shell of each Mn(II) completed with two bpy ligands resulting in a distorted octahedral geometry. The complex has crystallographic P-1 symmetry, the symmetric center being coincident on the C1—C1^#^ bond of the oxalate bridge, and the distance between the two metal centres is 5.583 (1) Å. Finally, intermolecular π···π stacking interactions (interplanar distances between bpy rings = 3.739 (1) Å) (Janiak, 2000), assemble the binuclear cations into a three-dimensional supermolecular array as shown in Fig. 2. There are extensive interionic C-H···O interactions between the cations and between the cation and anion.

### Experimental

An ethanol solution of 0.1562 g (1.0 mmol) bpy in 5 ml EtOH was added dropwise to a stirred aqueous solution of 0.1809 g (0.5 mmol) Mn(ClO_4_)_2_.6H_2_O and 0.0674 g Na_2_C_2_O_4_ (0.5 mmol) in 10 ml H_2_O. After stirring about 30 min, the faint yellow filtrate (pH = 8.98) was subsequently allowed to stand at 318.15 K. Four days later, yellow needle crystals were obtained.

### Refinement

H atoms bonded to C atoms were placed in their geometrically calculated positions and refined using the riding model, with C–H distances 0.93Å and *U*_iso_(H) = 1.2 *U*_eq_(C). Three of the four perchlorate oxygen's were disordered over two conformations with occupancies of 0.852 (6) and 0.148 (6)and were constrained to be tetrahedral.

### Crystal data

**Table:**

[Mn_2_(C_2_O_4_)(C_10_H_8_N_2_)_4_](ClO_4_)_2_·C_10_H_8_N_2_	*Z* = 1
*M**_r_* = 1177.72	*F*(000) = 602
Triclinic, *P*1	*D*_x_ = 1.494 Mg m^−^^3^
Hall symbol: -P 1	Mo *K*α radiation, λ = 0.71073 Å
*a* = 9.4959 (19) Å	Cell parameters from 12924 reflections
*b* = 11.974 (2) Å	θ = 3.1–27.5°
*c* = 12.183 (2) Å	µ = 0.66 mm^−^^1^
α = 98.87 (3)°	*T* = 293 K
β = 102.73 (3)°	Needle, yellow
γ = 98.89 (3)°	0.18 × 0.15 × 0.08 mm
*V* = 1309.3 (4) Å^3^	

### Data collection

**Table:**

Rigaku R-AXIS RAPID diffractometer	5884 independent reflections
Radiation source: fine-focus sealed tube	2595 reflections with *I* > 2σ(*I*)
graphite	*R*_int_ = 0.071
Detector resolution: 0 pixels mm^-1^	θ_max_ = 27.5°, θ_min_ = 3.1°
ω scans	*h* = −12→11
Absorption correction: multi-scan (*ABSCOR*; Higashi, 1995)	*k* = −15→15
*T*_min_ = 0.888, *T*_max_ = 0.949	*l* = 0→15
12924 measured reflections	

### Refinement

**Table:**

Refinement on *F*^2^	Primary atom site location: structure-invariant direct methods
Least-squares matrix: full	Secondary atom site location: difference Fourier map
*R*[*F*^2^ > 2σ(*F*^2^)] = 0.072	Hydrogen site location: inferred from neighbouring sites
*wR*(*F*^2^) = 0.221	H-atom parameters constrained
*S* = 1.06	*w* = 1/[σ^2^(*F*_o_^2^) + (0.089*P*)^2^ + 0.5905*P*] where *P* = (*F*_o_^2^ + 2*F*_c_^2^)/3
5884 reflections	(Δ/σ)_max_ = 0.001
363 parameters	Δρ_max_ = 0.56 e Å^−^^3^
19 restraints	Δρ_min_ = −0.64 e Å^−^^3^

### Special details

**Table:**

Geometry. All esds (except the esd in the dihedral angle between two l.s. planes) are estimated using the full covariance matrix. The cell esds are taken into account individually in the estimation of esds in distances, angles and torsion angles; correlations between esds in cell parameters are only used when they are defined by crystal symmetry. An approximate (isotropic) treatment of cell esds is used for estimating esds involving l.s. planes.
Refinement. Refinement of F^2^ against ALL reflections. The weighted R-factor wR and goodness of fit S are based on F^2^, conventional R-factors R are based on F, with F set to zero for negative F^2^. The threshold expression of F^2^ > 2sigma(F^2^) is used only for calculating R-factors(gt) etc. and is not relevant to the choice of reflections for refinement. R-factors based on F^2^ are statistically about twice as large as those based on F, and R- factors based on ALL data will be even larger.

### Fractional atomic coordinates and isotropic or equivalent isotropic displacement parameters (Å^2^)

**Table:**

	*x*	*y*	*z*	*U*_iso_*/*U*_eq_	Occ. (<1)
Mn	0.77417 (7)	0.80535 (6)	0.43285 (6)	0.0524 (2)	
O1	1.0049 (3)	0.8552 (3)	0.5191 (3)	0.0646 (9)	
O2	0.8238 (4)	0.9833 (3)	0.4166 (3)	0.0656 (10)	
N1	0.5323 (4)	0.7980 (3)	0.3806 (3)	0.0528 (10)	
N2	0.7025 (4)	0.8552 (3)	0.5946 (3)	0.0518 (10)	
N3	0.8122 (4)	0.7399 (4)	0.2610 (3)	0.0575 (11)	
N4	0.7828 (5)	0.6195 (3)	0.4244 (4)	0.0612 (11)	
C1	1.0521 (5)	0.9651 (5)	0.5285 (4)	0.0559 (12)	
C2	0.4504 (5)	0.7646 (4)	0.2734 (4)	0.0615 (14)	
H2A	0.4965	0.7409	0.2165	0.074*	
C3	0.3028 (5)	0.7630 (5)	0.2415 (5)	0.0692 (16)	
H3A	0.2501	0.7391	0.1652	0.083*	
C4	0.2351 (6)	0.7973 (5)	0.3248 (5)	0.0683 (15)	
H4A	0.1347	0.7971	0.3060	0.082*	
C5	0.3167 (5)	0.8326 (4)	0.4381 (5)	0.0604 (14)	
H5A	0.2717	0.8569	0.4956	0.072*	
C6	0.4655 (4)	0.8313 (4)	0.4643 (4)	0.0455 (11)	
C7	0.5631 (5)	0.8696 (4)	0.5814 (4)	0.0467 (11)	
C8	0.5134 (6)	0.9182 (4)	0.6734 (4)	0.0596 (13)	
H8A	0.4160	0.9269	0.6633	0.072*	
C9	0.6107 (6)	0.9531 (5)	0.7790 (5)	0.0719 (15)	
H9A	0.5794	0.9863	0.8413	0.086*	
C10	0.7533 (6)	0.9394 (5)	0.7935 (4)	0.0730 (16)	
H10A	0.8200	0.9630	0.8650	0.088*	
C11	0.7952 (5)	0.8899 (5)	0.6999 (4)	0.0634 (14)	
H11A	0.8920	0.8797	0.7094	0.076*	
C12	0.8303 (5)	0.8059 (5)	0.1841 (5)	0.0707 (15)	
H12A	0.8101	0.8797	0.1959	0.085*	
C13	0.8775 (6)	0.7691 (6)	0.0886 (5)	0.0819 (18)	
H13A	0.8916	0.8176	0.0376	0.098*	
C14	0.9030 (7)	0.6600 (6)	0.0704 (5)	0.088 (2)	
H14A	0.9341	0.6329	0.0058	0.105*	
C15	0.8829 (6)	0.5888 (6)	0.1476 (5)	0.0798 (17)	
H15A	0.9001	0.5141	0.1354	0.096*	
C16	0.8367 (5)	0.6314 (5)	0.2431 (4)	0.0589 (13)	
C17	0.8178 (5)	0.5641 (4)	0.3330 (4)	0.0607 (13)	
C18	0.8351 (7)	0.4510 (5)	0.3256 (6)	0.0823 (17)	
H18A	0.8578	0.4128	0.2612	0.099*	
C19	0.8190 (8)	0.3959 (6)	0.4123 (6)	0.101 (2)	
H19A	0.8333	0.3205	0.4082	0.121*	
C20	0.7821 (8)	0.4499 (6)	0.5054 (6)	0.104 (2)	
H20A	0.7685	0.4124	0.5648	0.124*	
C21	0.7657 (7)	0.5624 (5)	0.5081 (5)	0.0921 (19)	
H21A	0.7414	0.6008	0.5716	0.110*	
N5	0.4060 (6)	0.5170 (4)	0.1117 (4)	0.0831 (14)	
C22	0.4831 (6)	0.4685 (4)	0.0451 (4)	0.0628 (14)	
C23	0.5290 (6)	0.3666 (5)	0.0573 (5)	0.0717 (15)	
H23A	0.5821	0.3352	0.0089	0.086*	
C24	0.4953 (7)	0.3123 (6)	0.1416 (6)	0.097 (2)	
H24A	0.5254	0.2434	0.1514	0.116*	
C25	0.4184 (8)	0.3597 (6)	0.2100 (6)	0.100 (2)	
H25A	0.3943	0.3242	0.2679	0.120*	
C26	0.3756 (8)	0.4618 (6)	0.1930 (6)	0.105 (2)	
H26A	0.3225	0.4939	0.2410	0.127*	
Cl	0.83136 (16)	0.15993 (14)	0.10897 (12)	0.0850 (5)	
O3	0.9051 (4)	0.0736 (3)	0.0794 (3)	0.1224 (18)	
O4A	0.7113 (5)	0.1554 (5)	0.0196 (4)	0.234 (4)	0.852 (6)
O5A	0.9245 (6)	0.2664 (3)	0.1272 (6)	0.264 (5)	0.852 (6)
O6A	0.7863 (5)	0.1514 (5)	0.2073 (3)	0.132 (2)	0.852 (6)
O4B	0.6882 (5)	0.1108 (7)	0.1053 (9)	0.234 (4)	0.148 (6)
O5B	0.8299 (12)	0.2336 (6)	0.0324 (6)	0.264 (5)	0.148 (6)
O6B	0.9008 (10)	0.2202 (7)	0.2182 (5)	0.132 (2)	0.148 (6)

### Atomic displacement parameters (Å^2^)

**Table:**

	*U*^11^	*U*^22^	*U*^33^	*U*^12^	*U*^13^	*U*^23^
Mn	0.0525 (4)	0.0465 (4)	0.0593 (5)	0.0092 (3)	0.0215 (3)	0.0030 (3)
O1	0.0597 (19)	0.051 (2)	0.082 (2)	0.0115 (16)	0.0223 (17)	0.0052 (18)
O2	0.063 (2)	0.060 (2)	0.075 (2)	0.0095 (18)	0.0201 (18)	0.0123 (18)
N1	0.058 (2)	0.045 (2)	0.053 (2)	0.0108 (18)	0.0187 (19)	−0.0015 (18)
N2	0.046 (2)	0.058 (2)	0.051 (2)	0.0095 (18)	0.0127 (17)	0.0115 (19)
N3	0.055 (2)	0.064 (3)	0.056 (2)	0.015 (2)	0.0181 (19)	0.008 (2)
N4	0.088 (3)	0.044 (2)	0.059 (2)	0.014 (2)	0.035 (2)	0.006 (2)
C1	0.051 (3)	0.072 (3)	0.052 (3)	0.023 (2)	0.019 (2)	0.012 (2)
C2	0.060 (3)	0.065 (3)	0.051 (3)	0.007 (3)	0.014 (2)	−0.008 (3)
C3	0.052 (3)	0.075 (4)	0.067 (3)	0.007 (3)	−0.002 (3)	0.004 (3)
C4	0.050 (3)	0.063 (3)	0.089 (4)	0.011 (3)	0.014 (3)	0.014 (3)
C5	0.052 (3)	0.057 (3)	0.073 (3)	0.012 (2)	0.023 (3)	0.005 (3)
C6	0.046 (2)	0.037 (2)	0.055 (3)	0.0058 (19)	0.018 (2)	0.006 (2)
C7	0.052 (2)	0.038 (2)	0.053 (3)	0.005 (2)	0.022 (2)	0.009 (2)
C8	0.071 (3)	0.053 (3)	0.060 (3)	0.016 (2)	0.028 (3)	0.007 (2)
C9	0.101 (4)	0.067 (4)	0.056 (3)	0.019 (3)	0.039 (3)	0.008 (3)
C10	0.089 (4)	0.079 (4)	0.042 (3)	0.005 (3)	0.011 (3)	0.003 (3)
C11	0.058 (3)	0.072 (4)	0.057 (3)	0.009 (3)	0.006 (2)	0.017 (3)
C12	0.068 (3)	0.081 (4)	0.068 (4)	0.012 (3)	0.023 (3)	0.023 (3)
C13	0.090 (4)	0.098 (5)	0.060 (4)	0.014 (4)	0.024 (3)	0.021 (3)
C14	0.090 (4)	0.120 (6)	0.053 (3)	0.014 (4)	0.030 (3)	0.007 (4)
C15	0.089 (4)	0.080 (4)	0.068 (4)	0.016 (3)	0.031 (3)	−0.011 (3)
C16	0.059 (3)	0.062 (3)	0.056 (3)	0.014 (2)	0.021 (2)	0.002 (3)
C17	0.067 (3)	0.053 (3)	0.062 (3)	0.010 (3)	0.022 (3)	0.004 (3)
C18	0.112 (4)	0.055 (4)	0.087 (4)	0.024 (3)	0.041 (4)	0.004 (3)
C19	0.148 (6)	0.054 (4)	0.110 (5)	0.029 (4)	0.048 (5)	0.013 (4)
C20	0.174 (7)	0.058 (4)	0.095 (5)	0.021 (4)	0.062 (5)	0.024 (4)
C21	0.148 (5)	0.067 (4)	0.082 (4)	0.029 (4)	0.063 (4)	0.019 (3)
N5	0.129 (4)	0.060 (3)	0.076 (3)	0.022 (3)	0.055 (3)	0.015 (2)
C22	0.076 (3)	0.053 (3)	0.059 (3)	0.011 (3)	0.017 (3)	0.011 (2)
C23	0.087 (4)	0.063 (4)	0.074 (4)	0.023 (3)	0.031 (3)	0.015 (3)
C24	0.123 (5)	0.079 (4)	0.112 (5)	0.035 (4)	0.055 (4)	0.037 (4)
C25	0.152 (6)	0.072 (4)	0.095 (5)	0.020 (4)	0.062 (4)	0.031 (4)
C26	0.161 (6)	0.076 (5)	0.103 (5)	0.036 (4)	0.071 (5)	0.021 (4)
Cl	0.1096 (11)	0.0957 (11)	0.0654 (9)	0.0500 (9)	0.0353 (8)	0.0140 (8)
O3	0.126 (3)	0.125 (4)	0.112 (4)	0.070 (3)	0.023 (3)	−0.023 (3)
O4A	0.306 (8)	0.325 (10)	0.080 (4)	0.248 (8)	−0.035 (5)	0.000 (5)
O5A	0.403 (10)	0.076 (5)	0.367 (11)	−0.036 (6)	0.322 (9)	−0.038 (6)
O6A	0.196 (6)	0.161 (6)	0.080 (3)	0.065 (5)	0.088 (4)	0.044 (3)
O4B	0.306 (8)	0.325 (10)	0.080 (4)	0.248 (8)	−0.035 (5)	0.000 (5)
O5B	0.403 (10)	0.076 (5)	0.367 (11)	−0.036 (6)	0.322 (9)	−0.038 (6)
O6B	0.196 (6)	0.161 (6)	0.080 (3)	0.065 (5)	0.088 (4)	0.044 (3)

### Geometric parameters (Å, °)

**Table:**

Mn—O1	2.156 (3)	C12—C13	1.372 (8)
Mn—O2	2.159 (3)	C12—H12A	0.9300
Mn—N4	2.226 (4)	C13—C14	1.359 (9)
Mn—N1	2.228 (4)	C13—H13A	0.9300
Mn—N3	2.245 (4)	C14—C15	1.387 (8)
Mn—N2	2.249 (4)	C14—H14A	0.9300
O1—C1	1.301 (6)	C15—C16	1.385 (7)
O2—C1^i^	1.233 (5)	C15—H15A	0.9300
N1—C2	1.329 (6)	C16—C17	1.483 (7)
N1—C6	1.356 (5)	C17—C18	1.381 (7)
N2—C7	1.339 (5)	C18—C19	1.352 (9)
N2—C11	1.349 (6)	C18—H18A	0.9300
N3—C12	1.338 (6)	C19—C20	1.359 (9)
N3—C16	1.348 (6)	C19—H19A	0.9300
N4—C21	1.337 (7)	C20—C21	1.375 (8)
N4—C17	1.340 (6)	C20—H20A	0.9300
C1—O2^i^	1.233 (5)	C21—H21A	0.9300
C1—C1^i^	1.514 (9)	N5—C26	1.330 (8)
C2—C3	1.365 (6)	N5—C22	1.333 (7)
C2—H2A	0.9300	C22—C23	1.376 (7)
C3—C4	1.361 (7)	C22—C22^ii^	1.489 (10)
C3—H3A	0.9300	C23—C24	1.365 (8)
C4—C5	1.390 (7)	C23—H23A	0.9300
C4—H4A	0.9300	C24—C25	1.342 (9)
C5—C6	1.382 (6)	C24—H24A	0.9300
C5—H5A	0.9300	C25—C26	1.377 (9)
C6—C7	1.480 (6)	C25—H25A	0.9300
C7—C8	1.391 (6)	C26—H26A	0.9300
C8—C9	1.370 (7)	Cl—O6A	1.371 (3)
C8—H8A	0.9300	Cl—O6B	1.378 (4)
C9—C10	1.365 (7)	Cl—O5B	1.378 (4)
C9—H9A	0.9300	Cl—O4A	1.380 (3)
C10—C11	1.370 (7)	Cl—O3	1.382 (3)
C10—H10A	0.9300	Cl—O4B	1.383 (4)
C11—H11A	0.9300	Cl—O5A	1.391 (3)
			
O1—Mn—O2	77.71 (13)	N2—C11—H11A	118.5
O1—Mn—N4	91.32 (15)	C10—C11—H11A	118.5
O2—Mn—N4	161.81 (14)	N3—C12—C13	122.7 (6)
O1—Mn—N1	163.26 (13)	N3—C12—H12A	118.6
O2—Mn—N1	92.48 (14)	C13—C12—H12A	118.6
N4—Mn—N1	101.34 (15)	C14—C13—C12	118.3 (6)
O1—Mn—N3	94.71 (14)	C14—C13—H13A	120.9
O2—Mn—N3	92.97 (15)	C12—C13—H13A	120.9
N4—Mn—N3	73.29 (16)	C13—C14—C15	120.4 (6)
N1—Mn—N3	99.37 (14)	C13—C14—H14A	119.8
O1—Mn—N2	93.27 (13)	C15—C14—H14A	119.8
O2—Mn—N2	91.44 (14)	C16—C15—C14	118.6 (6)
N4—Mn—N2	103.77 (15)	C16—C15—H15A	120.7
N1—Mn—N2	73.22 (14)	C14—C15—H15A	120.7
N3—Mn—N2	171.55 (14)	N3—C16—C15	120.8 (5)
C1—O1—Mn	111.3 (3)	N3—C16—C17	116.3 (4)
C1^i^—O2—Mn	114.1 (3)	C15—C16—C17	122.8 (5)
C2—N1—C6	118.3 (4)	N4—C17—C18	120.8 (5)
C2—N1—Mn	124.4 (3)	N4—C17—C16	116.0 (4)
C6—N1—Mn	117.3 (3)	C18—C17—C16	123.2 (5)
C7—N2—C11	118.4 (4)	C19—C18—C17	119.8 (6)
C7—N2—Mn	116.4 (3)	C19—C18—H18A	120.1
C11—N2—Mn	124.2 (3)	C17—C18—H18A	120.1
C12—N3—C16	119.2 (4)	C18—C19—C20	120.6 (6)
C12—N3—Mn	123.9 (4)	C18—C19—H19A	119.7
C16—N3—Mn	116.3 (3)	C20—C19—H19A	119.7
C21—N4—C17	118.1 (5)	C19—C20—C21	117.2 (6)
C21—N4—Mn	124.2 (4)	C19—C20—H20A	121.4
C17—N4—Mn	117.5 (3)	C21—C20—H20A	121.4
O2^i^—C1—O1	123.9 (4)	N4—C21—C20	123.6 (6)
O2^i^—C1—C1^i^	117.6 (6)	N4—C21—H21A	118.2
O1—C1—C1^i^	118.5 (6)	C20—C21—H21A	118.2
N1—C2—C3	124.2 (5)	C26—N5—C22	116.4 (5)
N1—C2—H2A	117.9	N5—C22—C23	123.0 (5)
C3—C2—H2A	117.9	N5—C22—C22^ii^	115.8 (6)
C4—C3—C2	118.0 (5)	C23—C22—C22^ii^	121.2 (6)
C4—C3—H3A	121.0	C24—C23—C22	119.0 (5)
C2—C3—H3A	121.0	C24—C23—H23A	120.5
C3—C4—C5	119.7 (5)	C22—C23—H23A	120.5
C3—C4—H4A	120.1	C25—C24—C23	119.1 (6)
C5—C4—H4A	120.1	C25—C24—H24A	120.5
C6—C5—C4	119.2 (5)	C23—C24—H24A	120.5
C6—C5—H5A	120.4	C24—C25—C26	118.9 (7)
C4—C5—H5A	120.4	C24—C25—H25A	120.5
N1—C6—C5	120.7 (4)	C26—C25—H25A	120.5
N1—C6—C7	115.7 (4)	N5—C26—C25	123.6 (6)
C5—C6—C7	123.6 (4)	N5—C26—H26A	118.2
N2—C7—C8	121.5 (4)	C25—C26—H26A	118.2
N2—C7—C6	116.5 (4)	O6B—Cl—O5B	109.7 (4)
C8—C7—C6	122.0 (4)	O6A—Cl—O4A	109.8 (3)
C9—C8—C7	118.7 (5)	O6A—Cl—O3	112.2 (3)
C9—C8—H8A	120.7	O6B—Cl—O3	109.6 (4)
C7—C8—H8A	120.7	O5B—Cl—O3	109.6 (4)
C10—C9—C8	120.4 (5)	O4A—Cl—O3	109.2 (3)
C10—C9—H9A	119.8	O6B—Cl—O4B	109.6 (4)
C8—C9—H9A	119.8	O5B—Cl—O4B	109.4 (4)
C9—C10—C11	118.1 (5)	O3—Cl—O4B	109.0 (4)
C9—C10—H10A	120.9	O6A—Cl—O5A	108.0 (3)
C11—C10—H10A	120.9	O4A—Cl—O5A	108.3 (3)
N2—C11—C10	122.9 (5)	O3—Cl—O5A	109.2 (3)
			
O2—Mn—O1—C1	6.6 (3)	C3—C4—C5—C6	−0.6 (8)
N4—Mn—O1—C1	171.9 (3)	C2—N1—C6—C5	−1.4 (7)
N1—Mn—O1—C1	−48.6 (7)	Mn—N1—C6—C5	177.9 (3)
N3—Mn—O1—C1	98.6 (3)	C2—N1—C6—C7	−178.8 (4)
N2—Mn—O1—C1	−84.2 (3)	Mn—N1—C6—C7	0.4 (5)
O1—Mn—O2—C1^i^	−7.8 (3)	C4—C5—C6—N1	1.2 (7)
N4—Mn—O2—C1^i^	−61.8 (6)	C4—C5—C6—C7	178.5 (4)
N1—Mn—O2—C1^i^	158.5 (3)	C11—N2—C7—C8	−0.5 (7)
N3—Mn—O2—C1^i^	−101.9 (3)	Mn—N2—C7—C8	−169.4 (3)
N2—Mn—O2—C1^i^	85.3 (3)	C11—N2—C7—C6	179.5 (4)
O1—Mn—N1—C2	145.5 (5)	Mn—N2—C7—C6	10.6 (5)
O2—Mn—N1—C2	92.1 (4)	N1—C6—C7—N2	−7.3 (6)
N4—Mn—N1—C2	−76.0 (4)	C5—C6—C7—N2	175.2 (4)
N3—Mn—N1—C2	−1.4 (4)	N1—C6—C7—C8	172.6 (4)
N2—Mn—N1—C2	−177.2 (4)	C5—C6—C7—C8	−4.8 (7)
O1—Mn—N1—C6	−33.7 (7)	N2—C7—C8—C9	0.8 (7)
O2—Mn—N1—C6	−87.2 (3)	C6—C7—C8—C9	−179.1 (4)
N4—Mn—N1—C6	104.7 (3)	C7—C8—C9—C10	−0.5 (8)
N3—Mn—N1—C6	179.4 (3)	C8—C9—C10—C11	−0.2 (9)
N2—Mn—N1—C6	3.6 (3)	C7—N2—C11—C10	−0.2 (8)
O1—Mn—N2—C7	162.3 (3)	Mn—N2—C11—C10	167.8 (4)
O2—Mn—N2—C7	84.5 (3)	C9—C10—C11—N2	0.5 (8)
N4—Mn—N2—C7	−105.6 (3)	C16—N3—C12—C13	−2.0 (8)
N1—Mn—N2—C7	−7.7 (3)	Mn—N3—C12—C13	168.8 (4)
N3—Mn—N2—C7	−37.0 (12)	N3—C12—C13—C14	1.7 (9)
O1—Mn—N2—C11	−6.0 (4)	C12—C13—C14—C15	−0.6 (9)
O2—Mn—N2—C11	−83.7 (4)	C13—C14—C15—C16	−0.1 (9)
N4—Mn—N2—C11	86.2 (4)	C12—N3—C16—C15	1.3 (7)
N1—Mn—N2—C11	−175.9 (4)	Mn—N3—C16—C15	−170.3 (4)
N3—Mn—N2—C11	154.8 (9)	C12—N3—C16—C17	178.6 (4)
O1—Mn—N3—C12	−88.0 (4)	Mn—N3—C16—C17	7.1 (5)
O2—Mn—N3—C12	−10.1 (4)	C14—C15—C16—N3	−0.2 (8)
N4—Mn—N3—C12	−177.9 (4)	C14—C15—C16—C17	−177.4 (5)
N1—Mn—N3—C12	82.9 (4)	C21—N4—C17—C18	0.2 (8)
N2—Mn—N3—C12	111.3 (10)	Mn—N4—C17—C18	175.6 (4)
O1—Mn—N3—C16	83.1 (3)	C21—N4—C17—C16	−179.5 (5)
O2—Mn—N3—C16	161.0 (3)	Mn—N4—C17—C16	−4.2 (6)
N4—Mn—N3—C16	−6.8 (3)	N3—C16—C17—N4	−2.0 (7)
N1—Mn—N3—C16	−106.0 (3)	C15—C16—C17—N4	175.3 (5)
N2—Mn—N3—C16	−77.6 (11)	N3—C16—C17—C18	178.2 (5)
O1—Mn—N4—C21	86.3 (5)	C15—C16—C17—C18	−4.4 (8)
O2—Mn—N4—C21	138.6 (5)	N4—C17—C18—C19	−1.1 (9)
N1—Mn—N4—C21	−82.7 (5)	C16—C17—C18—C19	178.7 (6)
N3—Mn—N4—C21	−179.2 (5)	C17—C18—C19—C20	1.7 (11)
N2—Mn—N4—C21	−7.4 (5)	C18—C19—C20—C21	−1.5 (11)
O1—Mn—N4—C17	−88.7 (4)	C17—N4—C21—C20	0.0 (10)
O2—Mn—N4—C17	−36.4 (7)	Mn—N4—C21—C20	−175.0 (5)
N1—Mn—N4—C17	102.3 (4)	C19—C20—C21—N4	0.6 (11)
N3—Mn—N4—C17	5.8 (3)	C26—N5—C22—C23	0.5 (9)
N2—Mn—N4—C17	177.6 (3)	C26—N5—C22—C22^ii^	−179.7 (6)
Mn—O1—C1—O2^i^	173.2 (4)	N5—C22—C23—C24	−0.4 (9)
Mn—O1—C1—C1^i^	−5.2 (7)	C22^ii^—C22—C23—C24	179.9 (6)
C6—N1—C2—C3	0.9 (8)	C22—C23—C24—C25	0.1 (10)
Mn—N1—C2—C3	−178.4 (4)	C23—C24—C25—C26	0.0 (11)
N1—C2—C3—C4	−0.2 (9)	C22—N5—C26—C25	−0.4 (10)
C2—C3—C4—C5	0.1 (8)	C24—C25—C26—N5	0.2 (12)

Symmetry codes: (i) −*x*+2, −*y*+2, −*z*+1; (ii) −*x*+1, −*y*+1, −*z*.

### Hydrogen-bond geometry (Å, °)

**Table:**

*D*—H···*A*	*D*—H	H···*A*	*D*···*A*	*D*—H···*A*
C5—H5A···O1^iii^	0.93	2.61	3.356 (6)	138.
C5—H5A···O2^iv^	0.93	2.44	3.243 (6)	144.
C10—H10A···O3^v^	0.93	2.63	3.484 (6)	153.
C18—H18A···O5A	0.93	2.46	3.360 (8)	162.
C23—H23A···O4A	0.93	2.64	3.309 (7)	129.
C11—H11A···O6B^vi^	0.93	2.52	3.373 (10)	152.
C14—H14A···O5B^vii^	0.93	2.72	3.243 (12)	117.
C18—H18A···O5A	0.93	2.46	3.360 (8)	162.
C18—H18A···O5B	0.93	3.18	4.069 (10)	161.
C19—H19A···O6B	0.93	2.69	3.225 (9)	117.

Symmetry codes: (iii) *x*−1, *y*, *z*; (iv) −*x*+1, −*y*+2, −*z*+1; (v) *x*, *y*+1, *z*+1; (vi) −*x*+2, −*y*+1, −*z*+1; (vii) −*x*+2, −*y*+1, −*z*.
